# Genetic Disorders of Manganese Metabolism

**DOI:** 10.1007/s11910-019-0942-y

**Published:** 2019-05-14

**Authors:** S. Anagianni, K. Tuschl

**Affiliations:** 1 Department of Cell and Developmental Biology, University College London, Gower Street, WC1E 6BT, London, UK; 2 Department of Developmental Neurobiology, King’s College London, New Hunt’s House, Guy’s Campus, London, SE1 1UL UK; 3 UCL GOS Institute of Child Health, 30 Guilford Street, London,, WC1N 1EH UK

**Keywords:** SLC30A10, SLC39A14, SLC39A8, HMNDYT1, HMNDYT2, Manganese

## Abstract

**Purpose of Review:**

This article provides an overview of the pathogenesis, clinical presentation and treatment of inherited manganese transporter defects.

**Recent Findings:**

Identification of a new group of manganese transportopathies has greatly advanced our understanding of how manganese homeostasis is regulated *in vivo*. While the manganese efflux transporter SLC30A10 and the uptake transporter SLC39A14 work synergistically to reduce the manganese load, SLC39A8 has an opposing function facilitating manganese uptake into the organism. Bi-allelic mutations in any of these transporter proteins disrupt the manganese equilibrium and lead to neurological disease: Hypermanganesaemia with dystonia 1 (SLC30A10 deficiency) and hypermanganesaemia with dystonia 2 (SLC39A14 deficiency) are characterised by manganese neurotoxicity while SLC39A8 mutations cause a congenital disorder of glycosylation type IIn due to Mn deficiency.

**Summary:**

Inherited manganese transporter defects are an important differential diagnosis of paediatric movement disorders. Manganese blood levels and MRI brain are diagnostic and allow early diagnosis to avoid treatment delay.

## Introduction

Manganese (Mn) is one of the essential heavy metals required for normal growth and metabolism. Mn participates in a variety of physiological processes acting as a cofactor for multiple enzymes including transferases, hydrolases, lyases, isomerases, ligases and oxidoreductases [1, 2]. It is ubiquitously present in our diet and in the water supply. Foods rich in Mn include legumes, seafood, leafy green vegetables, rice, nuts and whole grains [3, 4]. Mn exists in a number of different oxidation states including Mn^2+^, Mn^3+^ and Mn^7+^ [5]. The most stable oxidation state of manganese is Mn^2+^, which has a pale pink colour and forms biologically relevant compounds such as Mn sulphate (MnSO_4_) and Mn chloride (MnCl_2_) [6].

Homeostasis of Mn levels in the body is tightly regulated through intestinal absorption and hepatobiliary secretion of the metal into the gastrointestinal tract [7]. Due to its ubiquity, dietary Mn deficiency has not been reported. Toxicity through overexposure on the other hand has been well documented in occupational exposure in miners, welders, battery manufacturers and ferroalloy workers [3, 8]. Excess Mn mainly accumulates in the basal ganglia, particularly the globus pallidus, causing a distinct extrapyramidal syndrome known as manganism. Symptoms include initial cognitive and psychiatric disturbances followed by a movement disorder resembling Parkinson’s disease with limb rigidity, dystonia and a characteristic high-stepping gait [3, 9]. Deposition of the paramagnetic metal in the brain causes distinct MRI brain appearances with pronounced hyperintensity of the globus pallidus on T1-weighted and hypointensity on T2-weighted images [10]. Other causes for acquired manganism include high concentrations of Mn in drinking water, parenteral nutrition and Mn contaminated ephedrone preparations [3, 9]. Impaired hepatobiliary excretion of Mn in end-stage liver disease similarly leads to Mn overload, a condition described as acquired hepatocerebral degeneration [11].

Under physiological conditions, mammalian brain Mn levels measure 5–14 ng Mn/mg protein (corresponding to 20–53 μM Mn). Neurotoxicity occurs when Mn levels increase 3-fold in the brain reaching 16–42 ng Mn/mg protein or 60–150 μM Mn [12]. Increased levels of Mn are toxic causing oxidative stress, impaired mitochondrial function and cell death [9].

Several transporters have been identified to play an important role in Mn homeostasis; however, how these transporters coordinate is still poorly understood. Uptake of Mn^2+^ into the cells can be facilitated by a number of membrane transporters such as the divalent metal transporter 1 (DMT1/SLC11A2), ZRT/IRT-like proteins ZIP8 (SLC39A8) and ZIP14 (SLC39A14), the dopamine transporter (DAT), calcium channels, choline and citrate transporters [3, 9, 13]. In the blood, Mn^2+^ is oxidised by caeruloplasmin to Mn^3+^ which binds to transferrin (Tf) and is subsequently internalised through transferrin/transferrin receptor (Tf/TfR)-mediated endocytosis. Within the endosome, Mn^3+^ is then reduced to Mn^2+^ and uptake into the cytoplasm occurs via the DMT1 transporter. Manganese efflux is facilitated by the membrane-localised transporters ferroportin (Fpn or SLC40A1) and the solute carrier family 30 member 10 (SLC30A10), both of which are able to directly export Mn from the cytosol [3, 9, 13, 14]. Within the cell, Mn is shuttled via a number of organelle-specific transporters including the secretory pathway Ca^2+^ ATPase 1 (SPCA1 or PMR1) at the Golgi, the ATPase13A2 (ATP13A2/PARK9) at the lysosome, ATP13A1 at the ER and mitoferrin-1 (Mfrn-1) at the mitochondria [15–18]. Iron (Fe) competes with Mn for binding and uptake at a number of transporters including the Tf/TfR complex, DMT1 and ferroportin. Thus, increasing the oral uptake of iron can be used as a treatment of hypermanganesaemia [19, 20].

The first hereditary disorder of Mn metabolism associated with Mn neurotoxicity was reported in 2012 [21••, 22••]. Mutations in SLC30A10 lead to a syndrome of hypermanganesaemia with dystonia, polycythaemia and chronic liver disease, now referred to as hypermanganesaemia with dystonia 1 (HMNDYT1) (OMIM #613280). Mainstay of treatment is chelation therapy with intravenous disodium calcium edetate (EDTA-CaNa_2_) in combination with iron supplementation [21••, 22••, 23–25]. In 2016, a similar hereditary Mn transporter defect, hypermanganesaemia with dystonia 2 (HMNDYT2) (OMIM #617013), was described that can be distinguished from SLC30A10 deficiency by absence of liver involvement and polycythaemia. Mutations in SLC39A14 lead to rapidly progressive dystonia with variable parkinsonism and other neurological signs with onset during infancy or early childhood. Chelation therapy with EDTA-CaNa_2_ has been attempted with some success [26••]. Both inherited Mn transporter defects share pathognomonic MRI brain appearances with hyperintensity on T1-weighted images of the globus pallidus and striatum, and the white matter of the cerebrum and cerebellum, midbrain, dorsal pons and medulla while the ventral pons is typically spared [21••, 22••, 26••].

On the other hand, mutations in SLC39A8, another Mn uptake transporter, were found to be associated with decreased blood Mn levels. Mn deficiency causes diminished activity of Mn-dependent enzymes such as the β-1,4-galactosyltransferase and Mn superoxide dismutase (MnSOD), leading to dysglycosylation, referred to as congenital disorder of glycosylation type IIn (CDG2N), as well as impaired mitochondrial function [27••, 28••, 29]. Affected individuals present as early as infancy with developmental delay, short statue, dwarfism, seizures, hypotonia, and dystonia. Treatment with oral Mn and galactose present possible treatment strategies [30•].

Mutations in other transporter proteins with affinity to Mn (i.e. ATP13A2, ATP13A1, DMT-1 and Fpn) have been described that may affect Mn homeostasis on subcellular level; however, blood manganese levels remain unaffected and there is no evidence of Mn deposition [18, 31–33]. Therefore, this review will concentrate on the Mn transporter defects caused by mutations in SLC30A10, SLC39A14 and SLC39A8 that have a pronounced effect on Mn levels *in vivo* (Table 1). The recent identification of these inherited primary Mn transporter defects has greatly advanced our understanding of how Mn homeostasis is maintained in humans. It has highlighted a distinct network of transporters required for the shuttling of Mn across the cell membrane that act synergistically to preserve stable Mn stores. Gaining insights into the function and regulation of these transporters and their role in the maintenance of Mn homeostasis is crucial to better understand manganese-induced pathologies and improve therapeutic paradigms. This may have wider implications for other neurodegenerative disorders with multifactorial aetiology such as Parkinson’s disease (PD) where metal dyshomeostasis is a key feature [3, 18, 35–38]. In particular, Mn has been shown to promote the oligomerisation and aggregation of alpha-synuclein, a hallmark of PD [3, 35]. Juvenile forms of PD caused by mutations in ATP13A2 and Parkin increase susceptibility to Mn toxicity [18, 38]. Mn neurotoxicity shares common neuropathological features with PD including oxidative stress, mitochondrial dysfunction and impaired autophagy. *In vitro* studies have shown that metal chelation has a neuroprotective effect and therefore might present a promising treatment avenue for neurodegenerative disorders [36].

**Table 1:** Characteristics of inherited Mn transporter defects. (+) Dystonia has been described in some affected individuals. (Reprinted from: Molecular Genetics and Metabolism, Vol 124/ 2, L.H. Rodan, M. Hauptman, A.M. D’Gama, A.E. Qualls, S. Cao, K. Tuschl, F. Al-Jasmi, J. Hertecant, S.J. Hayflick, M. Wessling-Resnick, E.T. Yang, G.T. Berry, A. Gropman, A.D. Woolf, P.B. Agrawal, Novel Founder Intronic Variant in SLC39A14 in Two Families Causing Manganism and Potential Treatment Strategies, 161–167, (2018), with permission from Elsevier) [34]

	HMNDYT1	HMNDYT2	CDG2N
Affected gene	*SLC30A10*	*SLC39A14*	*SLC39A8*
Blood Mn	↑	↑	↓
Neurological involvement			
Dystonia	+++	+++	(+)
Parkinsonism	+ (adult-onset, 1 family)	+	–
Seizures	–	–	+++
Cognitive impairment	Relatively spared	Relatively spared	Pronounced developmental delay
Systemic involvement	Liver disease	–	Short stature
	Polycythaemia		Dwarfism
	Depletion of iron stores		Deafness
			Liver disease
Brain MRI	Characteristic	Characteristic	Variable
	T1-hyperintensity of the globus pallidus and white matter, pathognomonic sparing of the ventral pons	T1-hyperintensity of the globus pallidus and white matter, pathognomonic sparing of the ventral pons	T2 hyperintensity of the basal ganglia Cerebral/cerebellar atrophy
	T2-hypointensity of the globus pallidus	T2-hypointensity of the globus pallidus	T2-hyperintensity basal ganglia
Treatment	Chelation therapy Iron supplementation	Chelation therapy	Mn supplementation Galactose

### HMNDYT1-SLC30A10 Deficiency

#### Clinical Presentation and Treatment

**Fig. 1:**
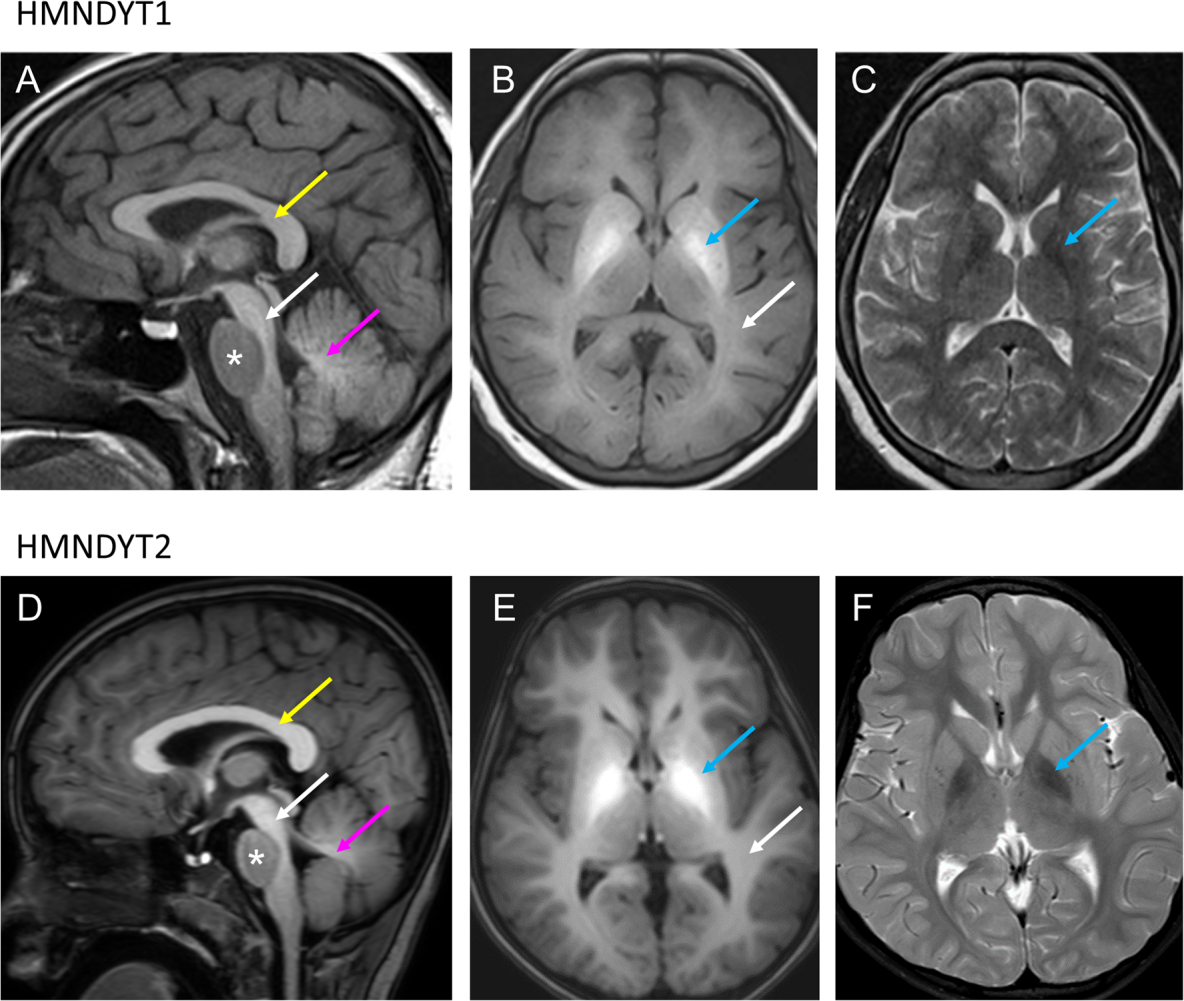
Characteristic appearances of Mn deposition in HMNDYT1 and HMNDYT2 on brain MRI. **a**–**c** Individual with HMNDYT1. **d**–**f.** Individual with HMNDYT2. **a, d** T1-weighted sagittal imaging showing hyperintensity of the white matter of the corpus callosum (yellow arrow), cerebellum (pink arrow) and the dorsal pons (white arrow) with characteristic sparing of the ventral pons (*). **b, e** T1-weighted transverse imaging showing hyperintensity of the globus pallidus (blue arrow) and cerebral white matter (white arrow) bilaterally. **c, f** T2-weighted transverse imaging showing hypointensity of the globus pallidus (blue arrow) bilaterally corresponding to T1-hyperintensities [22••, 26••]. (Reprinted from: Tuschl, K. *et al*. Syndrome of Hepatic Cirrhosis, Dystonia, Polycythemia, and Hypermanganesemia Caused by Mutations in SLC30A10, a Manganese Transporter in Man, Am. J. Hum. Genet, 90 (2012) 457–466; with permission from Elsevier) [22••]. (Reproduced from Tuschl, K. *et al*. Mutations in SLC39A14 Disrupt Manganese Homeostasis and Cause Childhood-Onset Parkinsonism-Dystonia, Nat Commun, 7: 11601 doi: 10.1038/ncomms11601 (2016); Creative Commons user license http://creativecommons.org/licenses/by/4.0/) [26••]

Hypermanganesaemia with dystonia 1 (HMNDYT1) caused by bi-allelic mutations in SLC30A10 was the first inherited manganese transporter defect described [21••, 22••]. To date, more than 30 patients have been reported (Table 2). Systemic Mn accumulation leads to a distinct syndrome of hypermanganesaemia, polycythaemia, dystonia, chronic liver disease (ranging from asymptomatic steatosis to cirrhosis with liver insufficiency) and depletion of iron stores. Blood Mn levels are dramatically raised, on average reported as ten times that of normal. On brain MRI, deposition of Mn is evident in the basal ganglia, particularly the globus pallidus and striatum with pronounced hyperintensity of T1-weighted imaging and corresponding hypointensity on T2-weighted imaging [21••, 22••, 23, 39–41]. There is additional involvement of the white matter of the cerebrum and cerebellum, midbrain, dorsal pons and medulla with a pathognomonic sparing of the ventral pons (Fig. 1). Clinically, the majority of patients present with dystonia during early childhood. Lower limb dystonia causes a characteristic high-stepping gait, also described as “cock walk gait”. White matter involvement can cause spasticity and pyramidal tract signs. A late onset form presenting as adult parkinsonism unresponsive to L-DOPA treatment has also been reported in one family so far [21••]. Cognition is typically normal. Histologically, severe neuronal loss in the globus pallidus and a vacuolated myelinopathy have been observed in the only case with postmortem examination reported to date [47]. Accumulation of Mn in the liver is hepatotoxic and leads to liver disease. However, the liver is not always clinically involved, at least at disease presentation, and liver disease might range from mild forms (steatosis) to severe forms (cirrhosis). Some patients have died due to liver cirrhosis. Polycythaemia has been reported in all patients and can be present prior to clinical symptoms. It has been suggested that Mn induces erythropoietin gene expression. Indeed, erythropoietin levels have been raised in some affected individuals [20]. As Mn and Fe compete for binding at several transporters, it is not surprising that iron stores are depleted in individuals with SLC30A10 mutations who show an increased total iron-binding capacity and a low ferritin [21••, 22••].

**Table 2:** Sequence changes in SLC30A10, SLC39A14 and SLC39A8 reported to date. *Nucleotide (c.) changes refer to the following transcripts: *SLC30A10* (NM_018713), *SLC39A14* (NM_015359.4) and *SLC39A8* (NM_022154.5). ^#^Amino-acid (p.) changes refer to the following protein isoforms: SLC30A10 (NP_061183), SLC39A14 (NP_056174.2) and SLC39A8 (NP_071437). ^1^Deletion of exon 3 and 4 caused by a large genomic deletion g.1qdel218,057,426_218,158,564 (GRCh36). ^2^Effect of splice site mutation not further examined

Mutation*	Amino acid change^#^	Number of affected individuals	Reference
**HMNDYT1 (SLC30A10)**			
c.[757_1696del]; [757_1696del]^1^		4	[22••]
c.[77T>C];	p.[Leu26Pro];	3	[39]
[77T>C]	[Leu26Pro]		
c.[90C>G];	p.[Tyr30*];	1	[39]
[90C>G]	[Tyr30*]		
c.[119A>C];	p.[Asp40Ala];	1	[39]
[119A > C]	[Asp40Ala]		
c.[122_124del];	p.[Ser41del];	1	[39]
[122_124del]	[Ser41del]		
c.[266T>C];	p.[Leu89Pro];	3	[22••]
[266T>C]	[Leu89Pro]		
c.[292_402del];	p.[Val98_Phe134del]; [Val98_Phe134del]	1	[22••]
[292_402del]			
c.[314_322del];	p.[Ala105_Pro107del]; [Ala105_Pro107del]	2	[22••]
[314_322del]			
c.[359G>A];	p.[Gly120Asp];	2	[40]
[359G>A]	[Gly120Asp]		
c.[460C>T];	p.[Gln154*];	1	[23]
[460C>T]	[Gln154*]		
c.[492del];	p.[Gly165Alafs*27];	3	[23]
[492del]	[Gly165Alafs*27]		
c.[496_553del];	p.[Ala166Glnfs*7];	1	[23]
[496_553del]	[Ala166Glnfs*7]		
c.[500T>C];	p.[Phe167Ser];	2	[21••]
[500T>C]	[Phe167Ser]		
c.[507del]; [507del]	p.[Pro170Leufs*22]; [Pro170Leufs*22]		
c.[585del];	p.[Thr196Profs*17];	1	[22••]
[585del]	[Thr196Profs*17]		
c.[765_767del];	p.[Val256del];	1	[22••]
[765_767del]	[Val256del]		
c.[780_782del];	p.[Iso260del];	2	[39]
[780_782del]	[Iso260del]		
c.[922C>T];	p.[Gln308*];	3	[22••, 41]
[922C>T]	[Gln308*]		
c.[957+1G>C];		2	[39]
[957+1G>C]^2^			
c.[1006C>T];	p.[His336Tyr];	3	[42]
[1006C>T]	[His336Tyr]		
c.[1046T>C];	p.[Leu349Pro];	1	[22••]
[1046T>C]	[Leu349Pro]		
c.[1235del];	p.[Gln412Argfs*26]; [Gln412Argfs*26]	2	[21••]
[1235del]			
**HMNDYT2 (SLC39A14)**			
c.[292T>G];	p.[F98V];	2	[26••]
[292T>G]	[F98V]		
c.[311G>T];	p.[Ser104Ile];	2	[43]
[311G>T]	[Ser104Ile]		
c.[313G>T];	p.[E105*];	2	[26••]
[313G>T]	[E105*]		
c.[367C>T];	p.[Gln123*];	1	[44]
[512G>A]	[Gly171Glu]		
c.[382C>T];	p.[R128W];	1	[45]
[382C>T]	[R128W]		
c.[477_478del];	p.[S160Cfs5];	1	[26••]
[477_478del]	[S160Cfs5]		
c.[751-9C>G];	p.[His251Profs26];	2	[34]
[751-9C>G]	[His251Profs26]		
c.[1136C>T];	p.[Pro379Leu];	1	[46]
[1136C>T]	[Pro379Leu]		
c.[1147G>A];	p.[G383R];	1	[26••]
[1147G>A]	[G383R]		
c.[1407C>G];	p.[N469K];	3	[26••]
[1407C>G]	[N469K]		
**CDG2N (SLC39A8)**			
c.[112G>C];	p.[Gly38Arg];	8	[27••]
[112G>C]	[Gly38Arg]		
c.[112G>C];	p.[Gly38Arg];	1	[28••]
[1019T>A]	[Ile340Asn]		
c.[338G>C];	p.[Cys113Ser];	2	[29]
[338G>C]	[Cys113Ser]		
c.[97G>A];	p.[Val33Met]	1	[28••]
[610G>T];	[Ser335Thr]		
[1004G>C]	[Gly204Cys]

Chelation with EDTA-CaNa_2_ has been effectively used to reduce Mn accumulation, treat neurological symptoms and prevent liver disease progression [20]. In most cases, Mn chelation leads to resolution of polycythaemia, normalisation of iron parameters and stabilisation of blood Mn levels. However, blood Mn levels often do not normalise but remain raised [20, 24, 40]. Effective reduction of the Mn load can be monitored on brain MRI indicated by a reduction of T1 hyperintensity. EDTA-CaNa_2_ is given intravenously as a 5 to 8 day course every 4 weeks. Close monitoring of calcium and other trace metal levels such as zinc (Zn), copper (Cu) and selenium (Se) is required to avoid adverse effects [25]. Some individuals have developed osteopenia and pathological fractures due to mobilisation of calcium from bone (personal communication, Claudio Melo de Gusmao).

While chelation with EDTA-CaNa_2_ is effective, the need of intravenous administration adds tremendously to the disease burden. Some case reports suggest that 2,3-dimercaptosuccinic acid as well as d-penicillamine present effective oral alternatives [39, 42]. It has yet to be determined whether chelation with either agent prevents disease progression long term. Iron supplementation alone has also been shown to improve clinical symptoms to some degree and reduce Mn levels [41].

In addition to chelation therapy, orally supplemented iron can act as a competitive ligand at Mn transporters leading to reduction of Mn absorption, stabilisation of Mn levels and further clinical improvement. Regular monitoring of iron parameters is required. The aim is to keep iron levels at the high end of normal without causing iron toxicity [21••, 22••, 23–25].

#### Pathogenesis of HMNDYT1

SLC3010 belongs to the SLC30 family of metal transporters. Members SLC30A1-8 or members SLC30A1 to SLC30A8 are expressed at the cell membrane where they are responsible for Zn efflux from the cytosol. Initially, SLC30A10 was also considered to be a Zn efflux transporter [12, 48]. However, studies in yeast have confirmed its crucial role in Mn transport [22••]. It has previously been shown that single amino acid changes are capable of changing the metal affinity of transporters [49]. For SLC30A10, the mechanism of metal coordination is substantially different to that of other members of the SLC30 family; however, the exact factors facilitating metal specificity are yet to be determined [12]. Studies by Leyva-Illades *et al.* have shown that wildtype SLC30A10 localises to the cell membrane where it mediates Mn efflux and protects against Mn-induced neurotoxicity without altering Zn levels or viability due to Zn toxicity. Some SLC30A10 mutations identified in patients have been shown to result in impaired efflux activity due to mislocalisation of the transporter in the endoplasmic reticulum and subsequent intracellular Mn accumulation [50].

SLC30A10 expression is specific to liver, gastrointestinal tract and brain [51]. Loss-of-function of SLC30A10 in both zebrafish and mice resemble the human phenotype with accumulation of Mn in blood, liver and brain [52•, 53]. Studies of tissue-specific SLC30A10 knockout mice have confirmed that under physiological conditions efflux activity of SLC30A10 is required in both liver and gastrointestinal tract, but not the brain, to maintain normal brain Mn levels. Hence, in addition to biliary excretion, manganese homeostasis is also maintained by luminal excretion of Mn by enterocytes. However, under increased Mn exposure SLC30A10 activity in the brain is further required to protect from Mn-induced neurotoxicity [52•]. Loss-of-function of Slc30a10 in zebrafish results in locomotor abnormalities that are associated with impaired GABAergic and dopaminergic signalling. Mutant zebrafish show increased expression of *atp2c1* encoding pmr1, a Golgi-expressed Mn transporter. It appears that *atp2c1* overexpression protects mutant embryos from Mn toxicity during early development uncovering a potential treatment target in individuals with Mn overload [53].

In SLC30A10 knockout mice, Mn accumulation in the thyroid gland also leads to pronounced hypothyroidism through Mn-induced inhibition of thyroxine production [54]. This has not been initially reported as a feature of HMNDYT1 in humans. However, we are aware that at least one patient with SLC30A10 mutations has hypothyroidism (personal unpublished observations). Whether the thyroid gland is always involved in this disease has yet to be determined.

### HMNDYT2-SLC39A14 Deficiency

#### Clinical Presentation and Treatment

In 2016, bi-allelic mutations in SLC39A14 were identified in individuals who presented with typical features of Mn neurotoxicity including rapidly progressive dystonia with variable signs of parkinsonism and T1-hyperintensity of the globus pallidus on brain MRI [26••]. While hypermanganesaemia was present in individuals with HMNDYT2, they did not show systemic features of Mn overload such as liver disease or polycythaemia. Blood levels of Fe, Zn and cadmium (Cd), divalent metals that can be transported by SLC39A14 in *in vitro* assays, were normal. Liver MRI was also normal suggesting absence of hepatic Mn accumulation. Overall, the onset of neurological symptoms appears to be earlier than observed in HMNDYT1 with some individuals being severely affected by hypotonia and dystonia within the first year of life [26••]. Axial hypotonia is then followed by dystonia, spasticity, dysarthria, bulbar dysfunction and signs of parkinsonism [44].

MRI brain appearances are identical to those seen in HMNDYT1 (Fig. 1). Postmortem examination of one affected individual showed marked neuronal loss in the globus pallidus, patchy loss of myelin associated with coarse vacuoles in the cerebral and cerebellar white matter, and axonal loss [26••]. To date, a total of 16 patients have been reported (Table 2) [26••, 34, 43–46].

Treatment with EDTA-CaNa_2_ according to the protocol used in HMNDYT1 has been attempted with marked success in one patient aged 5 years [44]. After 6 months of chelation therapy, neurological symptoms had improved and she regained the ability to walk [26••]. However, treatment in other patients has been less effective. While there is apparent mobilisation of Mn with increased urinary excretion of the metal and reduction of blood Mn, the neurological symptoms of these individuals did not improve dramatically [26••, 34, 43]. This may be due to differences in disease severity. It is likely that treatment is ineffective once neurodegeneration has progressed and becomes irreversible. In addition, the only individual with excellent treatment response carries mutations that solely affect one isoform of the transporter protein. Hence, the genotype might play a role in treatment response [26••]. Two oral chelants, 2,3-dimercaptosuccinic acid and d-penicillamine, were trialled in one patient but proved ineffective without increasing urinary Mn excretion [34].

In addition to Mn chelation, dietary restriction of Mn may also be of benefit. One affected individual was given 2 to 3 “Mn-free days” per week when she was solely fed Mn-depleted formula supplemented with a Mn-free multivitamin [34]. As she received EDTA-CaNa_2_ at the same time, it is not clear whether dietary Mn depletion had an additional effect on Mn levels and clinical symptoms.

#### Pathogenesis of HMNDYT2

SLC39A14 is part of the solute carrier 39 family that has been shown to facilitate uptake of Mn, Zn, Fe and Cd at the cell membrane [55–58]. Also known as Zrt, Irt-like protein 14 (ZIP14), it belongs to the LIV-1 subfamily and contains eight transmembrane domains, a histidine-rich motif (HXHXHX) as well as a metalloprotease motif (H/EEXPHEXGD) which is essential for metal transport [26••, 59, 60]. SLC39A14 mutations identified in affected individuals impair Mn uptake as demonstrated in HEK293 cells [26••].

The absence of hepatic Mn accumulation in affected individuals suggests that SLC39A14 is mainly required for Mn uptake into the liver for subsequent biliary excretion, and that the build-up of Mn in the brain occurs secondary due to impaired hepatic uptake of the metal. As observed in humans, loss of SLC39A14 function in mice and zebrafish leads to marked accumulation of Mn in the brain associated with impaired locomotor behaviour [26••, 61]. Hepatic uptake of Mn as well as excretion into the intestine is reduced in SLC39A14 knockout mice [61]. However, hepatocyte-specific SLC39A14 knockout mice do not develop a motor phenotype and have normal brain and serum Mn levels suggesting that Mn overload does not solely arise from impaired hepatic Mn uptake [62]. In mice, SLC39A14 is also expressed on the basolateral membrane of enterocytes where it may have an additional role of excreting Mn from the proximal intestine [61]. Little is known about the specific role of SLC39A14 in the brain where two of its isoforms are expressed. As Mn overload occurs in the brain as a result of SLC39A14 loss-of-function, Mn uptake must be facilitated through other Mn transporters than SLC39A14. However, it is possible that SLC39A14 is required at specific organelles where it is required for the maintenance of subcellular Mn homeostasis.

While SLC39A14 has been shown to transport a range of metals *in vitro* (Mn, Zn, Fe and Cd), loss of SLC39A14 function in humans, mice and zebrafish has little effect on metals other than Mn confirming that the main function of this transporter is the regulation of Mn homeostasis [26••, 61, 62].

### CDG2N-SLC39A8 Deficiency

#### Clinical Presentation and Treatment

The first inherited disorder associated with Mn deficiency was identified in 2015 and is caused by bi-allelic mutations in SLC39A8, a Mn uptake transporter (Table 2) [27••, 28••]. Systemic Mn deficiency leads to a multitude of symptoms including developmental delay, intellectual disability, failure to thrive, short stature, dwarfism, cranial asymmetry, seizures, hypotonia, dystonia, strabismus and deafness. Characteristically, blood Mn levels are low. Zn levels have been reported low in some patients, but normal in others. Affected individuals show an abnormal glycosylation pattern consistent with a type II congenital disorder of glycosylation. This is attributed to impaired function of Mn-dependent enzymes such as the β-1,4-galactosyltransferase required for the galactosylation of glycoproteins [27••, 28••, 29]. Patients can also present with Leigh-like mitochondrial disease characterised by elevated CSF lactate and abnormal respiratory chain enzymology associated with hyperintensity of the basal ganglia on T2-weighted MR imaging [29]. This is likely to occur due to dysfunction of the mitochondrial MnSOD. MRI brain imaging is unspecific; the majority of patients show cerebellar and/or cerebral atrophy [27••, 28••, 29].

Oral Mn supplementation has proven an effective treatment strategy. Two patients have been treated with oral Mn sulphate which led to significant clinical improvement of motor abilities and hearing and normalisation of enzyme functions [30•]. Prior to that, one individual was treated with galactose supplementation which resulted in normalisation of the glycosylation pattern [28••]. However, Mn supplementation appears to be the treatment of choice as it aims to resolve the primary Mn deficiency. Termination of galactose treatment while on Mn supplementation did not cause any clinical deterioration. To avoid Mn toxicity during Mn treatment, regular monitoring of blood Mn levels is required to ensure they are within the normal range together with MRI brain imaging to detect possible Mn deposition [30•].

#### Pathogenesis of CDG2N

SLC39A8, a member of the SLC39 family of metal-ion transporters, localises to the cell membrane and mediates influx of Mn as well as Zn, Fe and Cd [56, 57, 63]. Studies of SLC39A8 knockout mice confirmed its primary role in the regulation of Mn homeostasis. Loss-of-function in mice led to markedly reduced tissue Mn levels while levels of Zn and Fe remained unchanged. Liver-specific knockout also resulted in systemic Mn deficiency suggesting the liver to be the main target of function for SLC39A8. Corroborating this hypothesis, SLC39A8 was shown to localise to the apical surface of hepatocytes where it absorbs Mn from the bile, thereby reducing biliary Mn excretion [64•].

As an essential trace metal, Mn is required as a cofactor for a variety of enzymes and builds a constituent of metalloenzymes. Hence, the pathology of SLC39A8 deficiency can be explained by a multitude of enzyme deficiencies. Consistent with this hypothesis, SLC39A8 loss-of-function results in reduced activity of β-1,4-galactosyltransferase, arginase and MnSOD [28••, 64•, 65]. SLC39A8 mutations identified in affected individuals lead to reduced mitochondrial Mn levels associated with diminished activity of mitochondrial MnSOD and subsequent increase in oxidative stress [65].

## Conclusions

Identification of a new group of inherited Mn transporter defects caused by mutations in three solute carrier proteins, SLC30A10, SLC39A14, and SLC39A8 has helped us to understand how Mn homeostasis is regulated *in vivo*. Disturbance of this intricate network of transporters leads to detrimental changes in the body’s Mn load and subsequent disease with neurological (SLC39A14) or multiorgan involvement (SLC30A10, SLC39A8). A Mn blood level, a simple and cost-effective screening test, points towards diagnosis. Hence, routine neurological work-up of individuals with developmental delay or a movement disorder should include the determination of Mn blood levels. In parallel, diagnostic clues of Mn toxicity such as dystonia, parkinsonism, polycythaemia, liver disease and abnormal brain MRI, and that of Mn deficiency including dysglycosylation and mitochondrial disease should prompt genetic testing. Early diagnosis is critical to initiate appropriate treatment and avoid irreversible disease progression.
